# Intersemiotic translation of contracts into digital environments

**DOI:** 10.3389/frai.2022.963692

**Published:** 2022-10-11

**Authors:** Olimpia Giuliana Loddo, Andrea Addis, Giuseppe Lorini

**Affiliations:** ^1^Department of Law, University of Cagliari, Cagliari, Italy; ^2^Infora, Cagliari, Italy

**Keywords:** legal act, intersemiotic legal translation, legal effect, legal design, contract drafting, digital effect

## Abstract

An intersemiotic translation is any form of translation that involves at least two different semiotic codes; for example, the translation from words to images, to numerical code, or to non-verbal sounds. One of the most widespread examples of intersemiotic translation in the contemporary world is transposing natural language into machine language in digital environments. In this case, if the source text is a legal text, we encounter a particular type of intersemiotic translation, namely an intersemiotic legal translation in a digital environment. This paper will focus on the intersemiotic legal translation of contracts in digital environments, and is divided into two parts. In the first part (Section Ways of intersemiotically translating a contract using digital tools), we will analyze four possible uses of the intersemiotic translation of contracts in a digital context. In particular, we will highlight the technical characteristics of intersemiotic translation, its limitations, and its potential in different phases of contract management, namely the drafting of the document, the agreement, the archiving of the document, and the execution of contractual clauses. We will examine different digital tools that exploit intersemiotic translation, such as contract drafting tools and online platforms that allow for the conclusion of electronic contracts, document archiving in blockchains, and building smart contracts. When analyzing these uses of intersemiotic translation in the digital environment, we will highlight four types of output that can represent the product of intersemiotic translation in the digital environment: epistemic effects, legal effects, digital effects, and economic effects. In the second part (Section A tool for translating the contract intersemiotically), we will describe a hypothetical prototype that, in light of the four potential uses of intersemiotic translation, could represent a support tool to simplify the communication between professionals and clients through the drafting of legal documents with the aid of dynamic forms and, eventually, with the help of artificial intelligence (AI). Beyond facilitating the dialogue between legal professionals and their clients, we use interfaces to allow clients to create their own drafts of their documents and the lawyer to work on the drafts drawn up by the customer, correct them, and structure them in order to guarantee the validity of the document. The system can also be designed to archive legal documents and private deeds securely and entrust them to a professional by using blockchain technology and automating the execution of some contractual clauses *via* smart contract protocols.

## Introduction

An intersemiotic translation is any form of translation that uses at least two different semiotic codes, such as the translation from words to images, to numerical codes, or to non-verbal sounds. The topic of intersemiotic translation is currently the subject of lively debate^1^. Delving into the debate conducted by semioticians and translation theorists on the topic of intersemiotic translation is outside the scope of this paper; however, various authors have provided interesting perspectives for reflection on the purposes of our conceptual investigation. Specifically, they are all based on the concept of intersemiotic translation proposed by Jakobson^2^. The use of intersemiotic translation has mainly been investigated in semiotic art and literature studies. However, the relevance of intersemiotic translation in the legal field in general and, in the field of IT law in particular, is increasing rapidly.

Several examples of this type of intersemiotic translation apply to the drafting of contracts. Theorists of legal design encourage the use in the contract drafting of non-verbal semiotic tools in order to improve the comprehensibility of the contractual document and to transform it into a tool that is aimed at facilitating collaboration between the contractors, the function of which is not limited to the resolution of legal disputes (Barton et al., 2013). In particular, the use of pictures and drawings in contract drafting could be considered to be examples of “graphic translation.”

In this sense, intersemiotic translation can support the translation of abstract meanings; this element is particularly important concerning intersemiotic legal translations that focus mainly on legal concepts. However, the concept of intersemiotic translation is broader. The translation process that leads to the implementation of software is also intersemiotic translation. It is likely that this translation process is the most widespread form of intersemiotic translation in the contemporary world.

**Figure 1:**
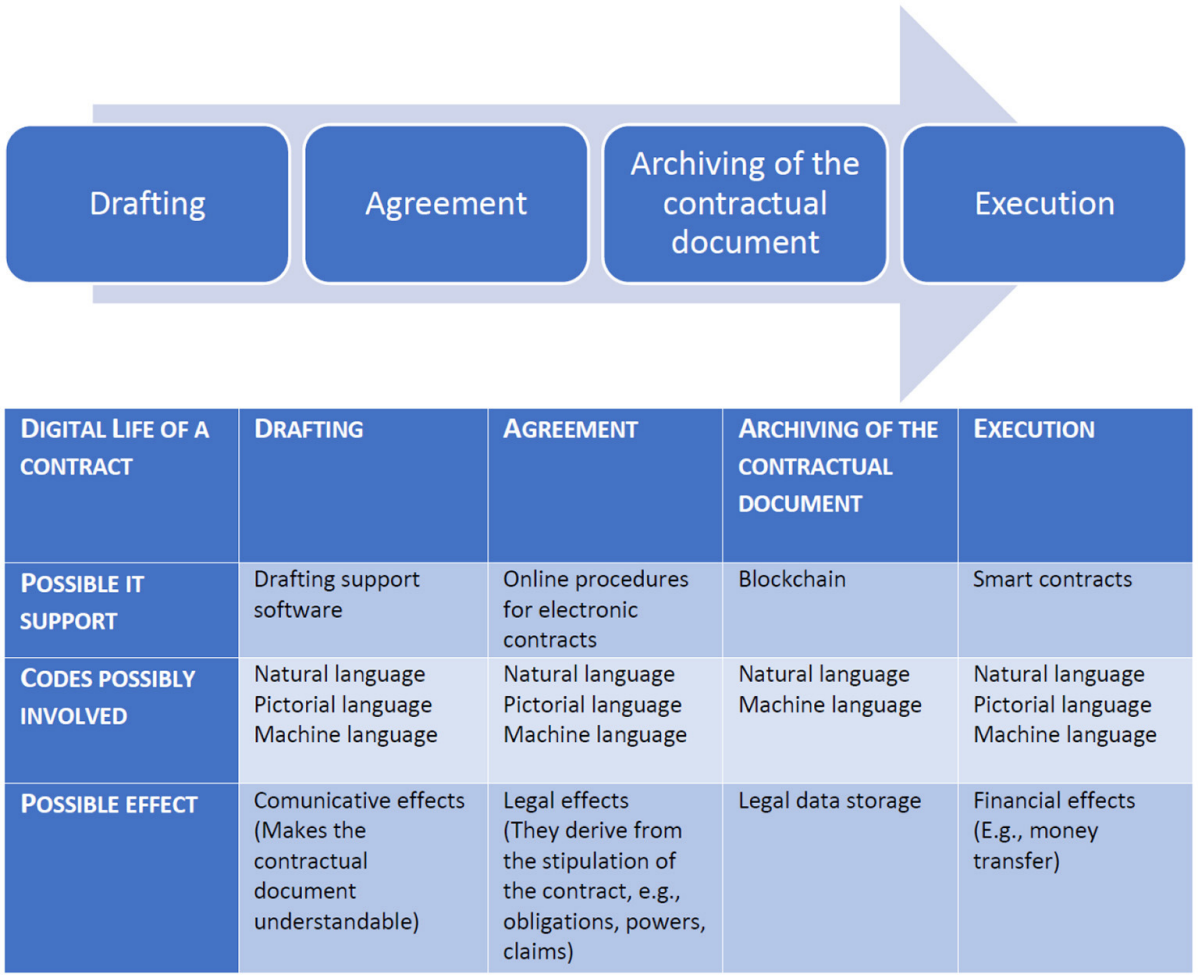
The digital management of a contract.

We will show how intersemiotic translation can operate in the different phases of a contract in a digital environment (Figure 1). A digital environment is an integrated communication environment in which digital devices are used to communicate and manage content and activities (Kulesz, 2017). In this context, we refer specifically to environments that facilitate the discovery of and search for information, people, and resources and which enable social and legal interactions (Mehdi, 2014).

Moreover, we note that techniques in intersemiotic translation can:

1. support the drafting of traditional contracts,
2. be the basis for the creation of platforms aimed at concluding electronic contracts,
3. be tools for archiving documents and making them unmodifiable, as in the case of blockchains, and
4. make the execution of contractual clauses automatic; for example, in smart contracts.

This paper is divided into two parts. In the first part (Section Ways of intersemiotically translating a contract using digital tools), we will analyze four possible uses of the intersemiotic translation of contracts in a digital environment. In particular, we will highlight the technical characteristics of intersemiotic translation, its limitations, and its potential in different phases of a contract's management: drafting, agreement, archiving, and execution. These different moments in the management of a contract are listed in this order for expository reasons. In fact, for instance, rental contracts are often registered when the tenant has already paid at least the first month's rent and the deposit. Alternatively, it is possible to decide to archive the contractual document after fulfilling its obligations. We will examine different digital tools that exploit different intersemiotic translations. In analyzing these uses of intersemiotic translation in the digital environment, we will highlight four types of output of intersemiotic translation in the digital environment, namely epistemic effects, legal effects, digital effects, and economic effects.

In the second part (Section A tool for translating the contract intersemiotically), we will describe a hypothetical prototype that, in light of the four potential uses of intersemiotic translation, could represent support that simplifies the work of professionals through tools that assist the drafting of legal documents with the aid of artificial intelligence (AI).

The support system could exploit machine learning algorithms to extend the knowledge base and to enhance the ontological representation, thus improving information retrieval and profiling algorithms, and assisting in the classification of social constructs and their symbolic representations in inherently complex legal systems.

This would facilitate the dialogue between legal professionals and their clients by creating an interface that allows clients to draft their documents with the support of AI and according to their needs, while simultaneously allowing the lawyer to work on the drafts drawn up by the customer, to correct them, and structure them in order to guarantee the validity of the document. A lawyer, acting as a platform manager, could also archive them and entrust them securely to the professional by using blockchain technology, automating the execution of some contractual clauses that are explicitly commissioned by the customers, and by binding some clauses *via* smart contract protocol.

## Ways of intersemiotically translating a contract using digital tools

Intersemiotic translation in the digital environment can occur during four different stages of the contractual document: the drafting of the contractual document, the conclusion of an agreement between the parties, the archiving of the document, and the fulfillment of the contractual obligations.

Without claiming to be exhaustive, this paragraph will provide an analysis of the different technologies that can occur in the four stages listed above, and will highlight their intersemiotic dimension, their limitations, and their potential. In particular, Section Intersemiotic translation using digital tools for the drafting of legal documents will focus on the drafting of the document; that is, the digital tools that use intersemiotic translation to compile the contractual document. Section Intersemiotic translation in the electronic contract concerns the formation of agreements in digital environments; Section Intersemiotic translation in archiving legal documents discusses the role of intersemiotic translation in document archiving, and will focus on archiving based on blockchain technologies. Finally, Section Intersemiotic translation in smart contracts refers to the execution of contractual clauses; that is, the obligations under the contract through telematic tools with particular reference to smart contracts. These forms of intersemiotic translation can flow into a single tool intended to facilitate the activity of lawyers.

### Intersemiotic translation using digital tools for the drafting of legal documents

In the common legal lexicon, there is a distinction between the contract and the contract form (Eisemberg, 2018, p. 521). The term “contract” refers to the legally enforceable agreement; this is a type of agreement in which the terms are based on prior negotiation, or at least discussion, among the parties in the agreement. By contrast, the expression “contract form” refers to a document that could vary materially (for example, it could be a digital or a paper document), or syntactically (it could include signs that differ in kind due to belonging to different semiotic systems), and modally (it could be aimed at different sensory apparatuses)^3^.

The mode of production of the contractual document is not rigidly established by the legal system, which asserts that some contracts have a written form or should be a public deed, but the instrument that is used for writing the contract form is not usually subject to discipline by the legislator. The legal system allows for flexibility in the drafting of contracts despite there being some constraints. This flexibility allowed for the introduction of the practice of drafting contractual documents based on extremely different models that could include forms of intersemiotic legal translation.

Two levels of intersemiotic translation can play a role in creating software for drafting a digital document. The first is intersemiotic translation which transposes natural language into machine language. In this case, this form of translation aims to create the conditions for the machine to be an intersemiotic translation tool and, in turn, to produce multimodal digital documents.

The second concerns the concrete use of the intersemiotic translation tool to draft legal documents.

When the user creates a document *via* a contract drafting platform, he or she can decide to use tools to illustrate the negotiation content based on non-linguistic semiotic systems.

It is an intersemiotic legal translation in both cases. The user who draws up a legal document *via* the platform makes layout and legal design choices that may involve the intersemiotic translation of contractual contents through graphics, images, drawings, timelines, and so forth^4^.

An intersemiotic translation that leads to the production of the software is also an intersemiotic legal translation because it must consider the properties of legal language; it must create models based on the regulations in force, which can affect the content of legal documents.

In recent years, some platforms have been developed to assist the lawyer to compile paper documents; for example, by making online forms available to users in order to allow them to enter data for the document they intend to draft.

Among these^5^, the Avokaado platform^6^, which is dedicated to contract management, is worthy of mention. The Avokaado start-up was created in 2016; this online platform allows clients to create drafts of standardized legal documents, such as contracts (Kerikmäe and Särav, 2017, p. 214). This start-up has the objective of encouraging the use of new technologies in the different phases of drafting contracts.

Digital platforms for drafting legal documents use AI to suggest the most appropriate formulations for the contractual clauses to the user^7^.

The interpretation of the contractual texts is often the product of elements characterizing the context in which the contract is stipulated, of commercial practices and customs; it presupposes information related to the cultural context in which the agreement is made. In this regard, the non-verbal semiotic codes adopted in legal drafting must be known to the contractors (for contract interpretation, see Mitchell, 2007; Burton, 2009).

Of interest, particularly in large companies, images, diagrams, tables, or other non-textual elements are used daily in managerial practice and, despite having contributed to the formation of contractual intentions, are traditionally separated or eliminated arbitrarily from the final contractual document (Mitchell, 2018).

Images, drawings and diagrams can contribute extensively to clarifying the meaning of a contractual document, and their use does not invalidate such documents, even those that, by law, must be in written form. In this sense, they *produce hermeneutic effects*.

It must be noted that these platforms operate within different legal systems. The meaning of a contractual term can vary considerably depending on the context and on the legal system in which it was stipulated. Therefore, the idea that we can imagine these platforms as possible substitutes for the support of a lawyer appears to be somewhat misleading. However, their usefulness is undeniable when they become a communication channel between the lawyer and the potential customer.

### Intersemiotic translation in the electronic contract

According to Casino et al. (2019, p. 1), “electronic contracts are contracts made by telegram, telephone, telex, fax, email, text message, and webpage. The categories of technology are not closed and may include contracts made by voice mail, Twitter, and the automated interaction of electronic agents. The characteristics that all these contracts have in common are that they are made remotely by electronic means.” Of interest, Blount differentiated between non-webpage electronic contracts (for example, those stipulated *via* email) and electronic webpage contracts (contracts that consist of a standard form that appears on an interactive website). The latter is particularly relevant to our analysis.

An electronic contract is an agreement between two individuals or companies to create *binding obligations*. For this reason, a contractual document drafted *via* digital legal support must theoretically be kept distinct from an electronic contract. Traditional contracts can be produced using digital tools without becoming electronic contracts.

Civil law scholars (e.g., Glatt, 1998; Blount, 2015; Tang, 2015; Wan et al., 2015) have questioned whether there is a difference between electronic and traditional contracts. From a legal point of view, an electronic contract presents all the fundamental elements of the contract, namely the agreement between the parties, the cause, and the object.

Electronic contracts are held to many of the same regulations as traditional contracts. For instance, the contract should clearly outline each party's responsibilities and dictate the requirements for full compliance. In other words, an electronic contract is an agreement by two or more parties to establish a legal patrimonial relationship that is usually stipulated *via* IT and transmitted *via* the internet.

Furthermore, it is now possible to stipulate an electronic contract respecting particular formal requirements; for example, by public deed. The main difference between a traditional paper contract and an electronic contract is the means of data transmission, which necessarily employs a process of intersemiotic translation^8^.

In electronic contracts, the intersemiotic translation process operates bidirectionally, as each contractor uses the platform not only to transpose signs belonging to different semiotic systems but to perform a legal act digitally.

This operation goes beyond the simple contractual drafting operation described in Section Intersemiotic translation using digital tools for the drafting of legal documents.

In this regard, the source text is the legal act that is carried out using a digital channel and is prepared or selected by the contracting parties or by a third party. This leads to the emergence of legal effects. As is the case for all legal acts, acts performed electronically may also be subject to conditions of invalidity deriving from the legal system to which the contracting parties refer.

These contracts are concluded by parties who are not in the same place.

Two possible options occur when the contract arises through a publicly accessible site. The website may contain a public offer or an invitation to offer. At present, the most popular mode of electronic contracts on websites consists of manifesting acceptance of one's contract proposal *via* “point and click,” or clicking on a “button” that appears on the screen (Marzotto, 2008); this is nothing more than an intersemiotic translation of an act of acceptance indicated by the signature. Since such contracts are made at a distance, the identification of the contracting parties is particularly relevant, and one of many forms of digital signatures can validate them. When a digital signature is not used, the validity of the electronic declaration must be assessed based on objective elements that allow for the verification of the contractor's identity^9^.

In contracts for purchasing software *via* the internet, the proposal, acceptance, payment of the price, and execution are instantaneous. Therefore, some rules based on the non-contemporaneity of these events are not applicable. It is important to note that, even if the execution of the obligations in an electronic contract may be immediate in some cases, the non-fulfillment of the contractual obligation remains possible.

### Intersemiotic translation in archiving legal documents

Legal documents are also translated into digital documents to allow for their conservation and archiving. One of the most interesting examples in this regard is the use of blockchain technology for this purpose (Galiev et al., 2018). Transactions are not validated by any central authority or trusted intermediary in blockchains; instead, all transactions are validated through cryptographic screening procedures.

In blockchains, all the users within the network can see all the transactions and can authenticate them. Once authenticated *via* the consent of the network users, the transactions are encoded with algorithms before being added to the blockchain, which is subsequently decrypted to produce the specified data, which are marked with a timestamp. Blockchain technology is thus essentially a form of distributed ledger technology (DLT).

Once coded and inserted into the blockchain, the contract cannot be modified, and operates according to its programmed instructions.

The homonymous technology was made famous by the spread of platforms aimed at exchanging cryptocurrencies. However, this technology also has other methods of use that have attracted some interest, both among lawyers and among IT scholars. A blockchain can be thought of as a distributed database organized as a list of ordered blocks in which the occupied blocks are immutable.

As can be seen from a recent literature analysis (Casino et al., 2019, p. 60), blockchains can have at least eight different main areas of application: financial, integrity verification, governance, internet of things (IoT), health, education, privacy and security, and business and industry. Of these, one with the greatest prospects for development falls within the field of governance: specifically, “[t]he emergence of the Internet of Agreements (IoA), which establishes the connection between digital contents (the internet) and real-world deals, contracts, or regulations, enables the next generation of digital commerce. Therefore, blockchain applications implement smart contracts to verify multiple types of operations” (Casino et al., 2019, p. 63).

The blockchain can determine the “digital” effect of the immutability of the data recorded in the chain; the inclusion of a legal document in the blockchain automatically determines its immutability. It will also not be possible to lose it; as it is difficult to conceive of an event that could result in the loss of data in all the chain's nodes, the document will always be recoverable and will not be falsifiable, nor will any form of counterfeiting be possible.

### Intersemiotic translation in smart contracts

Nick Szabo (1997), who is considered to have created the smart contract, defined it as “a computerized transaction protocol that executes the terms of a contract.”^10^

Based on the definition of the smart contract, it is possible to deduce that the protocols of smart contracts could be considered as a target text, whereas the source text is the set of contractual terms. As stated by Cervone et al. (2020), a “Smart Contract is considered a specific interpretation and translation (a codification) of its corresponding legal prose, which is the written expression of a mutual assent about the contractual terms (e.g., the considerations of a contract).”

The advantages of this technology were summarized by Szabo (1997), who stated: “Smart contracts reduce mental and computational transaction costs imposed by either principal, third parties, or their tools. […] This gives us new ways to formalize and secure digital relationships which are far more functional than their inanimate paper-based ancestors.”

A smart contract is a resource that should be considered carefully by lawyers who are more attentive to the application of new technologies to meet the needs of business and contract management (Corrales et al., 2019).

Some scholars have highlighted certain hermeneutical limitations of smart contracts; these limitations are linked to the fact that the negotiation program is, in fact, separate from the smart code, which makes it impossible to understand the nature of the transaction based solely on code^11^.

There are support tools that allow for the use of graphic symbols to draft smart contracts, and therefore to translate the smart contract interlinguistically from one programming language to another. Consider the Marlowe Playground, which is a tool for drafting smart contracts proposed by the Cardano platform, which allows the translation of a smart contract into Haskell, Javascript, Marlowe language, or even to use of the graphic symbols in the Blockly language^12^. However, unlike the traditional contractual text, the smart contract code is not meant to be interpreted by a human being but only executed by a machine.

In this regard, the most significant aspect of smart contracts is the intersemiotic translation from natural language to machine language. This translation results in an ontological change in the effects of the contract, which are no longer legal and mediated (since they need no longer to be implemented by another subject) but are financial and immediate (they occur automatically at the time of the program's execution). The software is capable of replacing the parties in fulfilling the contractual obligation. This alteration is reflected in the effects of the contract, which change from legal (obligations and claims) to become economic (money transfer).

More precisely, the drafting of a smart contract could be seen as the translation of a contractual clause from the deontic plan to the ontic plan. The clause becomes a program that will perform the service regardless of the parties' intervention. This alteration of the nature of the contractual clause has not gone unnoticed, and has raised many doubts among lawyers (Giancaspro, 2017; Governatori et al., 2018). In particular, Giancaspro (2017) pointed out that smart contracts also gave rise to several legal problems (also see Cuccuru, 2017). Automating the fulfillment of contractual obligations in smart contracts can be understood as a limit to contractual freedom. In fact, when the parties perform the contract according to their intentions at the time of formation, and their agreement was a valid and enforceable contract, they exercise their contractual freedom (Burton, 2009). However, if the clauses underlying the smart contract are subject to different interpretations by the contracting parties and it is necessary to ascertain whether the smart contract corresponds to the negotiating program, the freedom “from the contract” can be lost. In other words, if the planned performance in the smart contract, which is the intersemiotic translation of a contractual clause, differs from that described in the clause and requires the fulfillment of obligations that are not justified by the contract, a series of safeguarding measures that should be guaranteed to contractors are infringed.

For example, let us imagine a paid-for-performance contract that provides a money transfer *via* a smart contract for a performance in the real world. In the event of the non-performance of the service, how can it be exercised “*Exceptio non adimpleti contractus*” if the fulfillment is incorporated in an unchangeable algorithm that determines its automatic execution? Both smart contracts and other forms of the digitization and automation of contractual clauses say nothing about the legal and operational context within which the contract is made. There is no feedback regarding the automatic execution of contracts, and implementing revocations or renegotiations is impossible because the software becomes unchangeable once inserted into the blockchain^13^.

## A tool for translating the contract intersemiotically

The tool we are designing applies the different modes of intersemiotic contract translation described in the previous section, and aims to support both users who need to draft documents and service providers who need to draw up contracts. A further natural consequence of the service would be the storage of such documents in a certified repository. The tool offers the following types of services:

1. support in compiling legal documents by leveraging digital tools;
2. the archiving of documents by leveraging blockchain technology;
3. the automatic execution of certain contract clauses by leveraging smart contract technology.

The application is, in fact, an ecosystem in which different types of users coexist.

(i) An administrative department is in charge of developing, maintaining, and configuring the modules that constitute the system, and for enriching it with features as needs are detected over time.
(ii) Professionals, such as lawyers or labor consultants, are responsible for administering the platform from the editorial point of view; that is, enriching it with content, data structures, and information to support the processing of legal files as they change.
(iii) Platform users can exploit its advantages for initiating a case, drafting a contract, and entering into the same. These operations take place under the constant supervision of the legal department, which provides feedback, initiates practices, and returns the necessary information.

These users have different roles and permissions in terms of privileges within the application and, of course, in terms of interactions with it. For example, professionals would have the ability to access all the clients' documents, while the clients would only be able to view their accounts and documents.

The application would have the advantages of:

i. directing the client in the processing of legal paperwork, thus simplifying the law firm's bootstrap work;
ii. supporting the creation of clear, comprehensive, and non-redundant legal documents;
iii. leveraging the advantages of IT tools for contract certification (for example, through the blockchain) to facilitate registration processes;
iv. enabling the uploading of existing contracts and their structuring on the platform through the addition of dedicated parsers on known templates; and
v. exploiting translation tools in semi-structured metalanguage to give users (operators or end users) the ability to translate documents into other languages or media.

Some of these goals are achievable simply because of the in-built readiness of the selected technologies. Clearly, as was apparent in the previous chapter, this prototype is intended to refine and integrate technologies that have already been developed in existing tools and that are made available partly due to the nature of the open source technologies or services that are intended to be used. Examples of such features are as follows:

i. Agenda and Calendar: The ability to integrate appointment information, to manage an address book, appointment and deadline management, and staff meetings.
ii. To Do List: The software will create a list based on the status of practices still in progress, thus allowing the user to highlight work in progress or processes.
iii. Mail and Notification System: This would be triggered as a result of actions taken on the platform.
iv. Master and Practices: Customer communication and the automation of procedures.
v. Analysis Tools: These would facilitate strategic decisions or better planning for future activities.
vi. Improved Document Management: The use of templates, document reuse, and search functionality.
vii. Reporting and Financial Management.

### First access to the tool

The first way in which this prototype will be able to take advantage of intersemiotic translation in the contract is through the conclusion of an electronic contract^14^. In other words, the site will not merely be a showcase for a lawyer's firm, as is the case with many lawyers' sites at present, but will be a telematic section of a lawyer's firm. Clients will need to register and provide their information on the platform in order to enjoy the various services; in fact, it is essential for each user to have his or her own account in which to store drafts and documents.

Clearly, a section for communication will allow clients to contact the professional directly to ask for information, but it will still be necessary to register with the platform and create an individual account to access the compilation services. There would first need to be an electronic agreement between the professional and the potential client.

### Compilation of legal documents by exploiting digital tools

This is a service that allows the user to create a draft of the contract document. At certain stages of the draft creation process, a pool of microservices with intelligent features will suggest steps or notify the user about inconsistencies in the drafting. The tool is structured as follows:

CONTRACT TYPE → CLAUSE TEMPLATES → ILLUSTRATIONS AND GRAPHICS THAT CAN BE ASSOCIATED WITH THAT SPECIFIC CLAUSE.

The user will be able to choose the type of contract he or she wants to draft on a special menu, and can then move on to drafting the individual clauses. The AI will need to include a substantial number of templates in order for the user to have adequate support in the wording of the clauses. It will eventually be sufficient for the user to enter keywords to view a complete clause suggested by an intelligent search engine.

The managing professional of the platform cannot guarantee that the contract document is flawless at this stage. In fact, the content of the contract, although within the framework of legally well-structured wording, still represents user-defined content, which may omit certain conditions of invalidity established by the legal system.

Therefore, the support of a professional remains essential. However, a document completed by the contracting user(s) will enable the lawyer to have a clear idea of his or her clients' needs from the outset. In addition, the lawyer will have the opportunity to ensure appropriately structured, yet crisp, typo-free, standardized, and concise technical language. The use of graphics will add to the clarity of a document drafted in this way. The tool will offer users a semi-automatic compilation system that may include graphics at the users' discretion according to the strategies provided by the legal design. A pattern-recognition system may suggest one or more forms of graphic representations for each type of clause, and will exclude others.

### Document storage leveraging blockchain technology

A blockchain is a shared data structure that is “immutable,” and therefore generally incorruptible. It is defined as a digital ledger in which the entries are grouped into “blocks,” concatenated in chronological order, the integrity of which is guaranteed by the use of cryptography. Although its size is bound to increase over time, it is immutable in terms of the concept of “how much.” Once written its content can no longer be edited or deleted unless the entire process is invalidated.

Users may need to preserve documents, to make them unchangeable, and to have the assurance of archiving and storing them in a safe place. The document repository would automatically provide this functionality, but the recording and certification phase could be supported by placing the contract in a blockchain that is shared with a firm's clients.

### Smart contracts without digital coins

Users can entrust funds to a lawyer and create smart contracts; that is, they can create digital protocols that, upon the occurrence of certain conditions, produce a financial transaction automatically. In such a case, the clause will be executed automatically, and the digital economic effect will be the transfer from the user's wallet to that of another user on the platform.

Three possible use cases in which smart contract technology can be applied to the platform are presented below.

#### Service contract

Let us imagine two contractors who enter into a contract within the framework of this platform and decide that, when John performs service X, there will be a transfer from James' account to John's account. James deposits the amount due into the online wallet in advance. John executes the performance and notifies the platform operator. One can also assume the utilization and verification of a surety service.

#### Lease agreement

At the same time as entering into the lease agreement, the platform operator creates a smart contract that acts directly on the tenant's account to guarantee automatic payment of the monthly rent. In this case, however, an agreement with the bank is required.

#### A bequest

The testator binds a sum of money and creates a smart contract that will automatically transfer the sum of money to the testator's account when the succession occurs. Clearly, it will be the lawyer's responsibility to ensure (in advance) that the transaction does not harm the interests of the other heirs.

### Technologies

A number of prototypes were created within the macro architectural components of the application to support the analysis of the technologies involved in the development of an ecosystem of functionalities related to the intersemiotic translation of legal acts, and in order to experiment with the available technologies.

**Figure 2:**
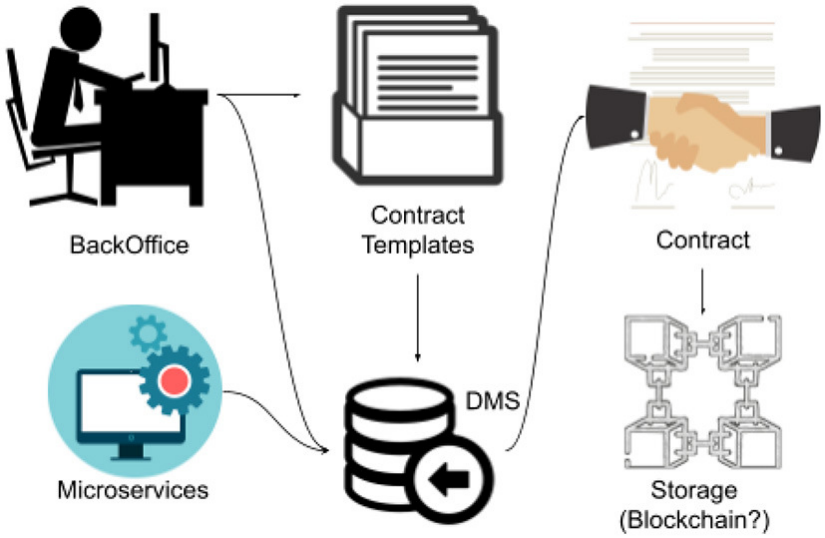
The proposed macro architecture.

The macro architecture (Figure 2) of the system consists of:

- A backoffice that is structured in such a way as to allow operators to define contract templates and their forms, and the users and professionals managing the platform to use them for contract drafting.
- A templating engine that, once a data entry has been made, allows it to be displayed according to the required specifications from the perspective of intersemiotic targeting.
- A pool of microservices for more complex functionalities, such as the translation or retrieval of information from a digital contract, other functionalities involving the use of machine learning algorithms or AI techniques, and the textual or semantic classification of the available repository.

Once the contract has been prepared, it can be reproduced using one or more of the existing templates and can possibly be saved on the blockchain.

#### Backoffice

Several solutions have been considered for the backoffice, including Spring Boot^15^ and Drupal^16^. On one hand, Spring Boot would allow microservices to be integrated in a more agile manner, and would lend itself better to binding with frontend interfaces on React technology^17^, thus providing support for the REST^18^ communication protocol; on the other hand, Drupal automatically provides all the tools for managing a repository of structured content and, above all, for complete and functional document management.

Drupal is in fact an open source content management framework (CMF) that is currently available in version 9. As one of the first frameworks for worldwide diffusion^19^, the use of Drupal has several advantages, among which are:

i Ease of presentation: Drupal allows for full correspondence with regulatory requirements (Accessibility, AgID, GDPR, and Cookies Law).
ii Security: Drupal includes features that mitigate all the risks indicated in the OWASP Top 10.
iii Reliability: Drupal is a mature solution; it has now been on the web for more than 15 years, is the result of the work of an experienced community, and has well-defined contribution rules.
iv Semantics: Drupal includes an articulate system of taxonomies for classifying and presenting content.
v Control: Administrators have extensive control over user roles and permissions, which are structured in such a way as to limit the actions they can take on the site to the bare minimum.
vi Editing: The Drupal backend is user-friendly, and versioning and content review systems are included. The publication of content will be organized according to a precise workflow of revision approval and publication *via* an editorial flow control system (Moderation).
vii Multilingual support: Drupal includes an articulated yet easy-to-use system for the management of multilingual content.
viii Integration: Using the appropriate modules, it is possible to integrate Drupal directly with the main components of the planned architecture, such as Elasticsearch. In addition, the full set of CRUD functionality is available via RESTful services, which allows other services to access the Drupal platform easily, thus ensuring seamless integration into the microservice architecture.

Drupal facilitates document management by being a good balance between a CMS and an Enterprise Content Management System (ECM), and by providing all backoffice functionality for managing structured bundles, as well as interfaces and widgets for handling all file formats.

With Drupal, it is possible to facilitate the drafting of contract clauses on the basis of a semi-structured form that can qualify conduct deontically (A is allowed, mandatory, or forbidden), or can establish incentives for certain conduct or set terms for performance, and can assign statuses (for example, the acquisition of status resulting from a career advancement within an employment contract).

In one of our prototypes, we experimented with drafting a norm consisting of a sequence of deontic situations that could be described according to their position in the deontic square (Conte, 1962, p. 87–88).

Note that the semi-structured compilation of the act allows for:

- Filling out the same document in different languages, since each entry can be localized.
- Once completed, the document will be shown to each user in his or her language (the website inherently provides localization tools).
- The document can be illustrated with graphical representations.

The use of Drupal greatly simplifies the logic-building part during the compilation of structured data that implement a contract type. In fact, this is done through extremely simple supporting backend interfaces compared to the *ad hoc* code structure, as is the case in Cicero^20^, a framework that is used to generate structured templates for any agreement.

#### Templating and front-end interfaces

At this stage, it was decided to adopt a Model-View-Controller (MVC) paradigm; that is, a software architectural pattern that is commonly used for developing user interfaces that divide the related program logic into three interconnected elements. This is done to separate internal representations of information from the ways in which information is presented to and accepted by the user.

This pattern, which was traditionally used for desktop graphical user interfaces (GUIs), became popular for designing web applications. Popular programming languages have MVC frameworks that facilitate the implementation of the pattern; specifically, the (M)odel component is the central component of the pattern, and is the application's dynamic data structure that is independent of the user interface. It directly manages the data, logic and rules of the application. The (V)iew is any representation of information, such as a chart, a diagram, or a table. Multiple views of the same information are possible, such as a bar chart for management and a tabular view for accountants. The (C)ontroller accepts input and converts it into commands for the model or view.

The model is responsible for managing the data of the application (the CMS); it receives user input from the controller (the CMS dispatchers). The view renders the presentation of the model in a particular format.

**Figure 3:**
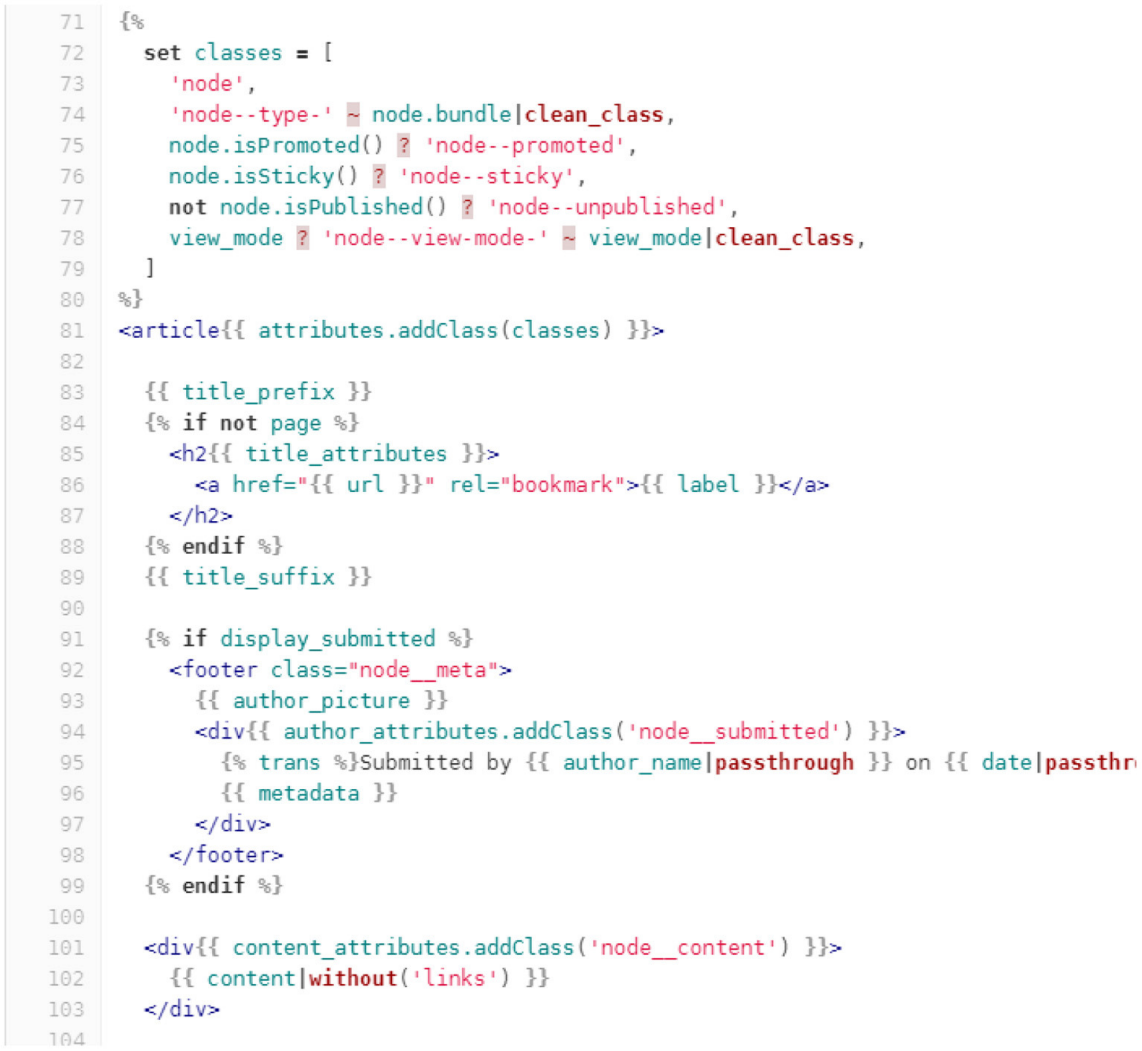
An example of a Twig representation of content.

In accordance with the MVC paradigm, it is possible to use Twig as the V(iew) (Figure 3) component because it is integrated into the Symphony Framework that is included in Drupal.

A visualization section of a generic node within Drupal is shown in the figure above. However, Drupal allows for the implementation of platforms and web applications in headless mode; that is, it allows for different technologies to be used for the frontend, such as the ReactJS web framework mentioned previously. It also allows for the possibility of developing advanced widgets, as in the graphical programming environment Scratch^21^ or the domain-specific language Marlowe^22^, which allows for the drafting of legal contracts (Lamela Seijas and Thompson, 2018).

#### Microservices and libraries

The prototype can integrate third-party libraries in order to develop microservices aimed at increasing the functionality of the platform, such as automatically importing compiled documents, the semantic classification of available contracts, and the semantic enrichment of search engines.

In this case, it would be ideal to use an object-oriented programming (OOP) paradigm based on the concept of “objects,” which can contain data and code. Languages such as Python, which make a huge number of AI libraries available, or a strictly typed language such as Java, would be ideal for the infrastructure of more delicate microservices, particularly for text processing.

In addition to “augmenting” the document repository, the microservice pool needs to be able to communicate with everything else on the platform *via* integration with all the supporting mechanisms, and possibly even relying on client-side analysis tools.

Visual Contract is an extremely trivial example that was developed to allow a text parser to draw elements on an HTML canvas from a contract interpreted by a Spring Boot microservice. The combination of these elements allows for a contract to be displayed based on certain parameters.

The code is implemented in an HTML/JS single page, with two.js^23^ libraries for the two-dimensional rendering of graphical objects and popper.js^24^ for the dynamic placement of informational tooltips. It implements visualization using two technologies for the graphical visualization of a known contract model (but using a general purpose schema).

**Figure 4:**
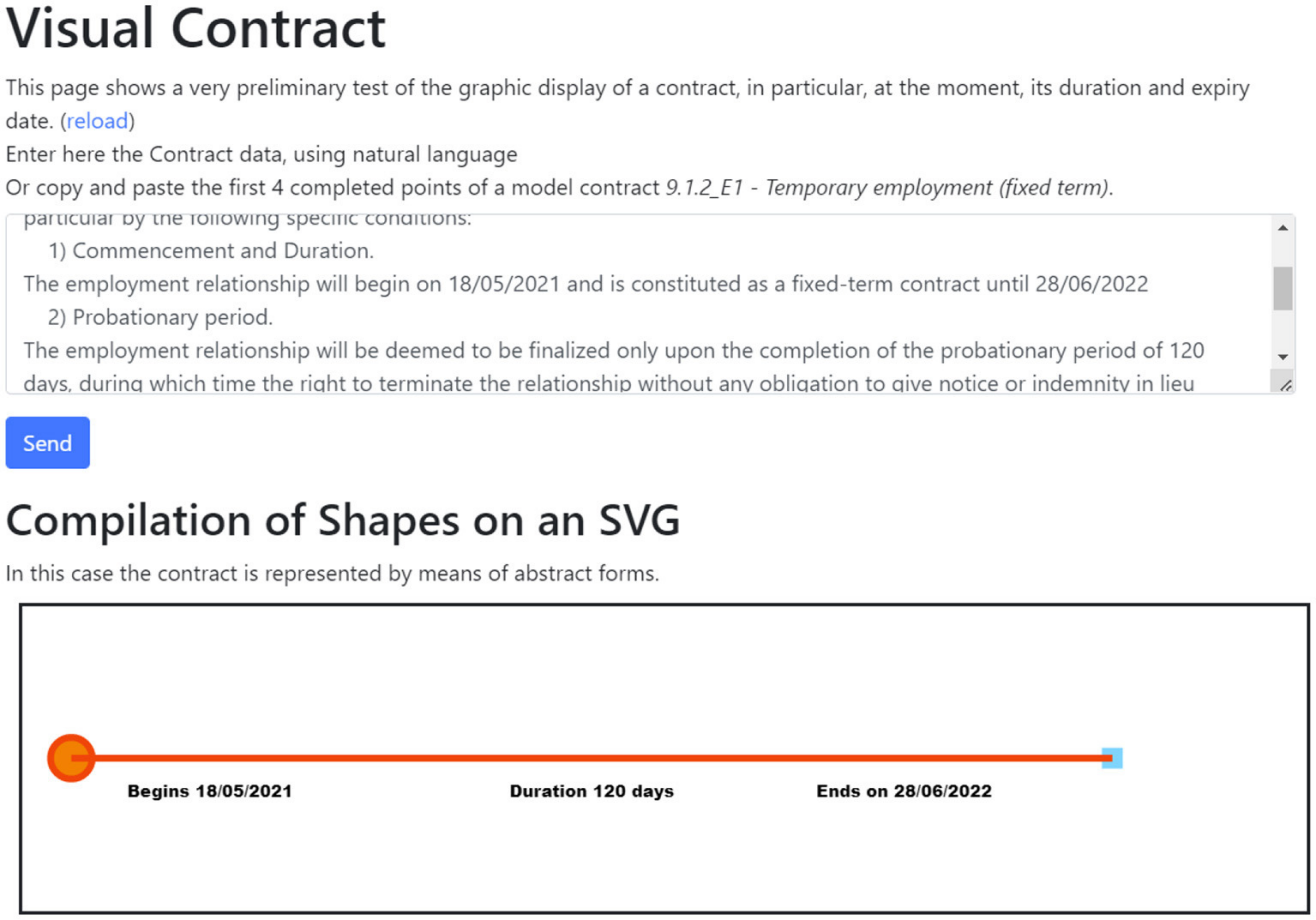
Example of the automatic translation of the timeline of a contract onto an SVG canvas.

**Figure 5:**
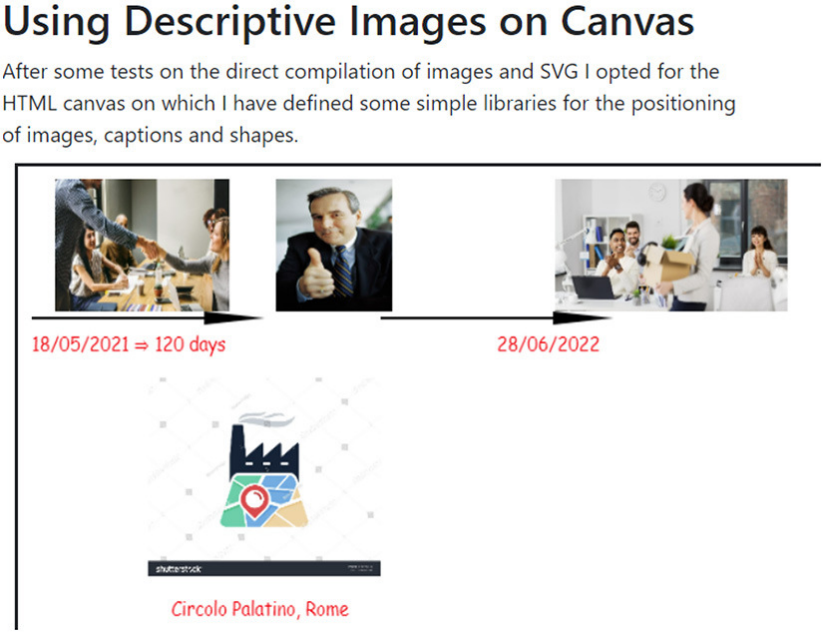
Example of automatic descriptive translation of the timeline of a contract.

In particular, the images in Figures 4, 5 show two examples of visualization, one with a graphics library on canvas, and the other with printed vector images on SVG using helpers for the arrangement of the images and the text on the canvas.

This is a simple example aimed at showing an intersemiotic translation from a predefined model to a graphical medium.

The use of chatbots cannot be ruled out, although one must be careful not to depersonalize the service (Adam et al., 2021) since conversational software agents, or chatbots, which are systems that are designed to communicate with human users by means of natural language and are often based on AI, frequently fail to meet customer expectations. In this regard, we have developed a simple prototype that attempts to demonstrate how natural language can be used to search legal databases.

Although some sites such as Normattiva^25^ provide a reference service, there is no way for developers to interface on structured, open code legal databases. Therefore, after autonomously compiling a database of articles from the first book of the Civil Code, we embarked on an experiment that entailed browsing the articles, which provided results both for the autocomplete mode and for answers to questions that were posed in natural language.

#### Storage

CMS provides a robust, reliable, and easy-to-use and expand document management and archiving mechanism. However, it is possible to certify and make the digitization of an agreement more secure by saving it in a blockchain.

**Figure 6:**
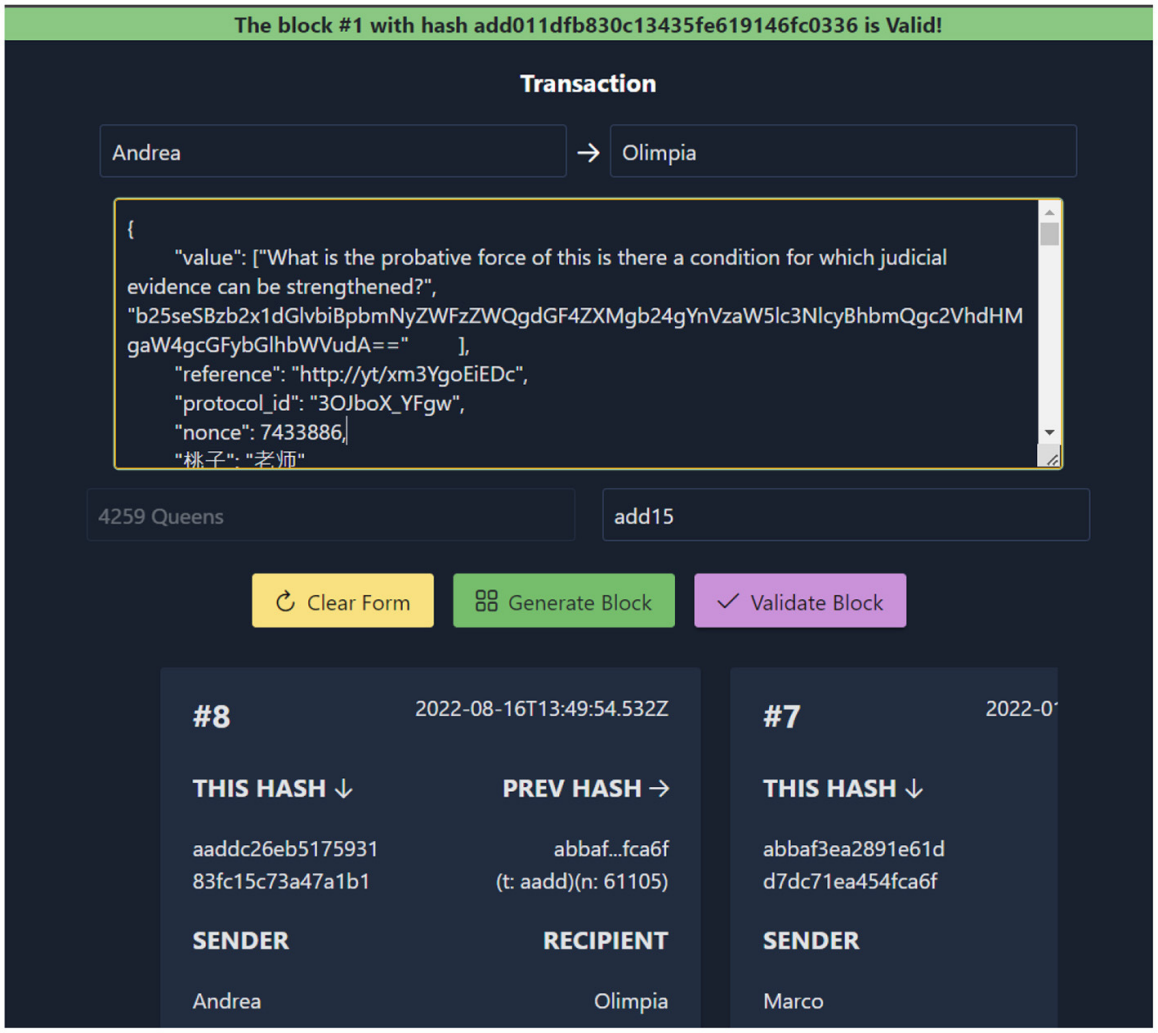
An example of completely client-side blockchain repository.

Creating a blockchain requires computational effort, the so-called proof of work, which we considered delegating to the client during the contract registration. In this regard, we experimented with ReactiveBlockChain^26^, which is a blockchain creation prototype that is written entirely in pure ReactJS; it was published *via* Netlify on an Elasticsearch DB as our own test repository (Figure 6).

Originally designed for educational purposes, this technology features a client-calculated proof of work; someone who places an item on the blockchain can determine its cost, and uses their browser's computational time to mine a new node.

The selection of the target allows a computational value to be assigned to the node, while the size of the content assigns a cost to storing it. The application also contains a validator of the nodes belonging to the chain. Of course, there are obvious security issues because the credentials are stored on the client-side code, but this is only a proof-of-concept tool, and this issue can be addressed by storing the credentials on a server-side middleware.

## Conclusions

In the field of contract drafting in the digital environment, intersemiotic legal translations represent an extremely powerful conceptual tool that can ease the work of professionals and their clients significantly. It can be used in various ways in different phases of contract management, and can limit both transaction costs and the asymmetry of information in negotiation exchanges. Nonetheless, legal translations need to be inserted into an architecture that considers both the legal and the economic dimensions of contractual exchanges. The intervention of a lawyer remains essential within this architecture, since all forms of intersemiotic translation entail changes in meaning which, if not monitored, impacting negatively on contractual freedom.

In this essay, we analyzed four methods for the intersemiotic translation of contracts in the digital environment, and identified their limitations and potential. In light of this theoretical analysis, we envisage the application of these methods in a single digital tool. This digital tool is able to meet the following needs:

i. simplifying the work of professionals by providing tools to support the drafting of legal documents with the aid of AI, which would be capable of extending the system knowledge base and enhance its ontological representation, thus improving information retrieval and profiling algorithms, as well as assisting in the classication of social constructs and symbolic representations that are inherently complex in legal systems;
ii. facilitating the dialogue between legal professionals and their clients by creating an interface that allows clients to create their own drafts of their documents with the support of AI and according to their needs, while also allowing the lawyer to work on the drafts drawn up by the customer, to correct them, and to structure them in order to guarantee the validity of the documents;
iii. the secure archiving of legal documents and private deeds by entrusting them to a professional using blockchain technology; and
iv. automating the execution of some contractual clauses that are explicitly commissioned by the customers by binding some clauses in the client's protocol through smart contracts.

The prototype is currently in the design phase and, for reasons related to the specificity of the legal language, will initially be disseminated in the Italian market. However, it is hoped that collaboration with foreign lawyers in an international project with the aim of creating a prototype that could have uses both in other countries and in international trade will become a reality in the future.

Therefore, in a subsequent phase of the realization, different versions of the product that are characterized by a structure similar to the one described will be produced, but which will present different content based on the potential stakeholders and the national regulatory system or the international reference standard.

## Author contributions

This article is the result of joint research undertaken by AA, OL, and GL. The final written version of Sections Introduction and Ways of intersemiotically translating a contract using digital tools can be attributed to OL, that of Section A tool for translating the contract intersemiotically to AA, Section Conclusions to GL. All authors contributed to the article and approved the submitted version.

## Conflict of interest

Author AA was employed by Infora. The remaining authors declare that the research was conducted in the absence of any commercial or financial relationships that could be construed as a potential conflict of interest.

## Publisher's note

All claims expressed in this article are solely those of the authors and do not necessarily represent those of their affiliated organizations, or those of the publisher, the editors and the reviewers. Any product that may be evaluated in this article, or claim that may be made by its manufacturer, is not guaranteed or endorsed by the publisher.

## Footnotes

^1^On intersemiotic translation see e.g., Plaza (1987), Torop (2000), Eco (2001), Dusi (2015), Queiroz and Aguiar (2015). For a recent reconstruction of the contemporary debate on intersemiotic translation, see Sütiste (2021).

^2^The expression “intersemiotic translation” was coined by the linguist Roman Jakobson (1959) in the paper *On Linguistic Aspects of Translations;* Jakobson, inspired by Peirce's (1955) theory of signs, proposed a distinction amongst three types of translation (intralingual, interlingual, and intersemiotic). According to Jakobson's definition, an intersemiotic translation is “an interpretation of verbal signs by means of signs of nonverbal sign systems”. Jakobson (1971), p. 235 later formulated a more general concept of intersemiotic transposition, namely “the transposition form one sign system to another”. On Roman Jackobson's triadic division of translation see Jia (2017).

^3^On this subject, the studies in Colette Brunschwig's work (See, e.g., Brunschwig, 2021) explore the multisensorization of legal contents, beginning with Kenney's theories concerning multisensory communication and multisensory media.

^4^For the use of images in the regulatory context, see Maynard (2017), Moroni and Lorini (2017, 2021), Lorini and Moroni (2020).

^5^Barton et al. (2022) mentioned several examples of these platforms. Among these examples, see *Contract Standards*. Available online at: https://www.contractstandards.com/ (accessed June 4, 2022); Juro,| The All-In-One Contract Automation Platform. Available online at: https://juro.com/ (accessed June 4, 2022); Lawgeex - Conquer Your Contracts. Available online at: https://www.lawgeex.com/ (accessed June 4, 2022); *Legal Sifter*. Available online at: https://www.legalsifter.com (accessed June 4, 2022). Open-source solutions are also developing, such as [Accord Project. *Accord Proj*. Available online at: https://accordproject.org/ (accessed June 4, 2022)].

^6^Avokaado. Contract Lifecycle Management Software | Legal Document Templates Avokaado. Available online at: https://avokaado.io/ (accessed February 19, 2022).

^7^Akorda - The Future of Legal Contracting. Available online at: https://www.akorda.com/ (accessed June 4, 2022); Ontra: *Contract Automation and Intelligence for Private Markets*. Available online at: https://www.ontra.ai/ (accessed June 4, 2022). By contrast, Legal Robot [Available online at: https://legalrobot.com/ (accessed June 4, 2022)] is a platform that is aimed at the interpretation and simplification of contracts [Legal Robot. Available online at: https://legalrobot.com/ (accessed June 4, 2022)].

^8^For example, as Glatt (1998, p. 44) mentioned, in the case of sending an email, the text is translated in binary code and “is broken into pieces by a computer program, the Transmission Control Protocol (TCP). According to the e-mail address given by the user these ‘packets' of data are ‘labeled' by another program, the Internet Protocol (IP). This enables the routers which receive the ‘packets' to send each of them in the fastest way to the addressee where they are restored to text”.

^9^As Tang (2015, p. 126) mentioned, “E-signatures may be present in different forms, including typing the signatory's name, scanned manual signature, a PIN or password, clicking the ‘I accept' icon, or digital signature”.

^10^Szabo defined the purchase from a humble vending machine as a primitive form of a “smart contract” as it involved the autonomous transfer of ownership of an asset, such as a sweet or a can of drink, upon receipt of a predetermined input (for example, money). Szabo also described a number of potential applications of smart contracts, including the automated transfer of digital properties (such as shares) upon the occurrence of a specific event; immobilization of the vehicle (where the vehicle would not work if the safety protocols provided for in the contract were not respected), and peer-to-peer property lending, whereby the loaned property would be returned to the lender if the borrower defaulted on specified conditions.

^11^The Riccardian contract (Grigg, 2004) was an early method for capturing the contractual relationship between contracting parties for supporting a later performance of the clauses. A Riccardian contract would appear, at least in part, to limit the hermeneutical problems of smart contracts. While a smart contract is executed based on a pro-forma set of instructions defined by parties, a Ricardian contract records all the details of the agreement in a machine-readable format and it is both machine-readable and human-readable. It combines both aspects of legal agreements and automated execution functionality by having a plain-text agreement that is readable by humans and a structured data agreement that is readable and executable by machines. Ricardian contracts include both executable code and other information bits that ensure human as well as computer readability. However, Ricardian contracts still lacked representational power due to the complex technicalities that traditional written contracts may contain in their legal prose.

^12^[*Marlowe Playground*. Available online at: https://marlowe-playground-staging.plutus.aws.iohkdev.io/#/ (accessed June 4, 2022)]. The Cardano platform provides the user with the possibility of drafting smart contracts using a simplified programming language (compared to traditional high-level languages) to realize its contractual intentions, which are converted into machine language. In this case, the machine is a tool that allows users to digitize their “contractual relationship” (Lamela Seijas and Thompson, 2018). In general, smart contracts are created *via* a simplified programming language that is specific to a particular domain. Not only are correctness and unambiguity essential formal properties, but also compliance with any legislation governing the matter involved in the transaction. The reliability, safety and security of the platform that executes the transactions are the main attributes.

^13^It is important not to confuse the concept of “smart contract” with that of “smart *legal* contract”. Of note, this concept appears in the consultation paper on Digital Assets [Available online at: https://www.lawcom.gov.uk/project/digital-assets/ (accessed August 16, 2022)] by the Law Commission of England and Wales that defines smart legal contracts as “A legally binding contract in which some or all of the contractual terms are defined in and/or performed automatically by a computer program”.

^14^See Section Intersemiotic translation in the electronic contract. Electronic contracts imply the use of electronic means to put distant parts in contact. The contract is made possible due to the electronic impulses exchanged between the terminals connected at a distance. As mentioned previously, the legal lexicon could be affected by national legal contracts. In this regard, it is worth mentioning that some French and Italian scholars use the term ‘telematic contract' to describe similar phenomena; see Stazi (2021).

^15^[Spring Boot – A solution for creating stand-alone, production-grade Spring-based Applications. Available online at: https://spring.io/projects/spring-boot (accessed June 7, 2022)].

^16^[Drupal - Open Source CMS. Available online at: https://www.drupal.org/home (accessed June 7, 2022)].

^17^[React – A JavaScript library for building user interfaces. Available online at: https://reactjs.org/ (accessed June 7, 2022)].

^18^[REST – *A Software Architectural Style That Describes A Uniform Interface Between Physically Separate Components*. Available online at: https://restfulapi.net/ (accessed June 7, 2022)].

^19^[Project usage overview. Available online at: https://www.drupal.org/project/usage (accessed June 7, 2022)].

^20^*Cicero Accord Proj*. Available online at: https://accordproject.org/projects/cicero/ (accessed June 7, 2022).

^21^[Scratch - A High-Level Block-Based Visual Programming Language And Website. Available online at: https://scratch.mit.edu/ (accessed June 7, 2022)].

^22^[*Marlowe Playground*. Available online at: https://marlowe-playground-staging.plutus.aws.iohkdev.io/#/ (accessed June 4, 2022)].

^23^[Two.js - A Two-Dimensional Drawing Api Geared Towards Modern Web Browsers. Available online at: https://two.js.org/ (accessed June 7, 2022)].

^24^[Popper.js - Tooltip and popover positioning engine. Available online at: https://popper.js.org/ (accessed June 7, 2022)].

^25^[Normattiva. Il portale della legge vigente - An Italian Government Website Containing the Rules of Italian Law. Available online at: https://www.normattiva.it/ (accessed June 7, 2022)].

^26^[Reactive BlockChain - A Clientside Blockchain Prototype. Available online at: https://reactblockchain.netlify.app/ (accessed June 7, 2022)].
